# Crystallization Inhibition and Solid-State Stability in High Drug Loaded Amorphous Drug-Polymer Dispersions Above the Overlap Concentration

**DOI:** 10.1007/s11095-026-04167-4

**Published:** 2026-08-03

**Authors:** Sichen Song, Ronald A. Siegel

**Affiliations:** 1 Department of Pharmaceutics, University of Minnesota, Minneapolis, MN 55455 USA; 2 School of Pharmacy, University of Wisconsin–Madison, Madison, WI 53705 USA; 3 Department of Biomedical Engineering, University of Minnesota, Minneapolis, MN 55455 USA

**Keywords:** amorphous solid dispersion, crystal growth, crystallization, nucleation, overlap concentration, solid state stability, viscosity

## Abstract

Amorphous solid dispersions (ASDs) have been shown to be an effective formulation strategy for improving apparent solubility, dissolution rate, and hence oral bioavailability of poorly aqueous soluble drugs. In this perspective, we review recent progress in understanding the role of polymer concentration, particularly the overlap concentration, c*, in governing crystallization of high drug loaded ASDs. The overlap concentration, c*, is a threshold above which adjacent polymer coils start to contact. Recent work has shown that when polymer concentration is below c*, the presence of the continuous polymer-free amorphous drug domain allows crystallization to proceed similarly to neat drug, resulting in limited inhibition. Above c*, formation of a homogeneous polymer rich matrix suppresses crystallization, primarily by delaying the first nucleation event. Representative case studies demonstrate the generality of this framework across diverse drug-polymer systems, although limitations arise for sufficiently high molecular weight polymers. Overall, the c*-guided rheological approach provides a mechanistic basis for rational polymer selection and optimization in high drug loaded, and therefore reduced overall volume, ASD formulations.

**Graphical Abstract:**

**Figure:**
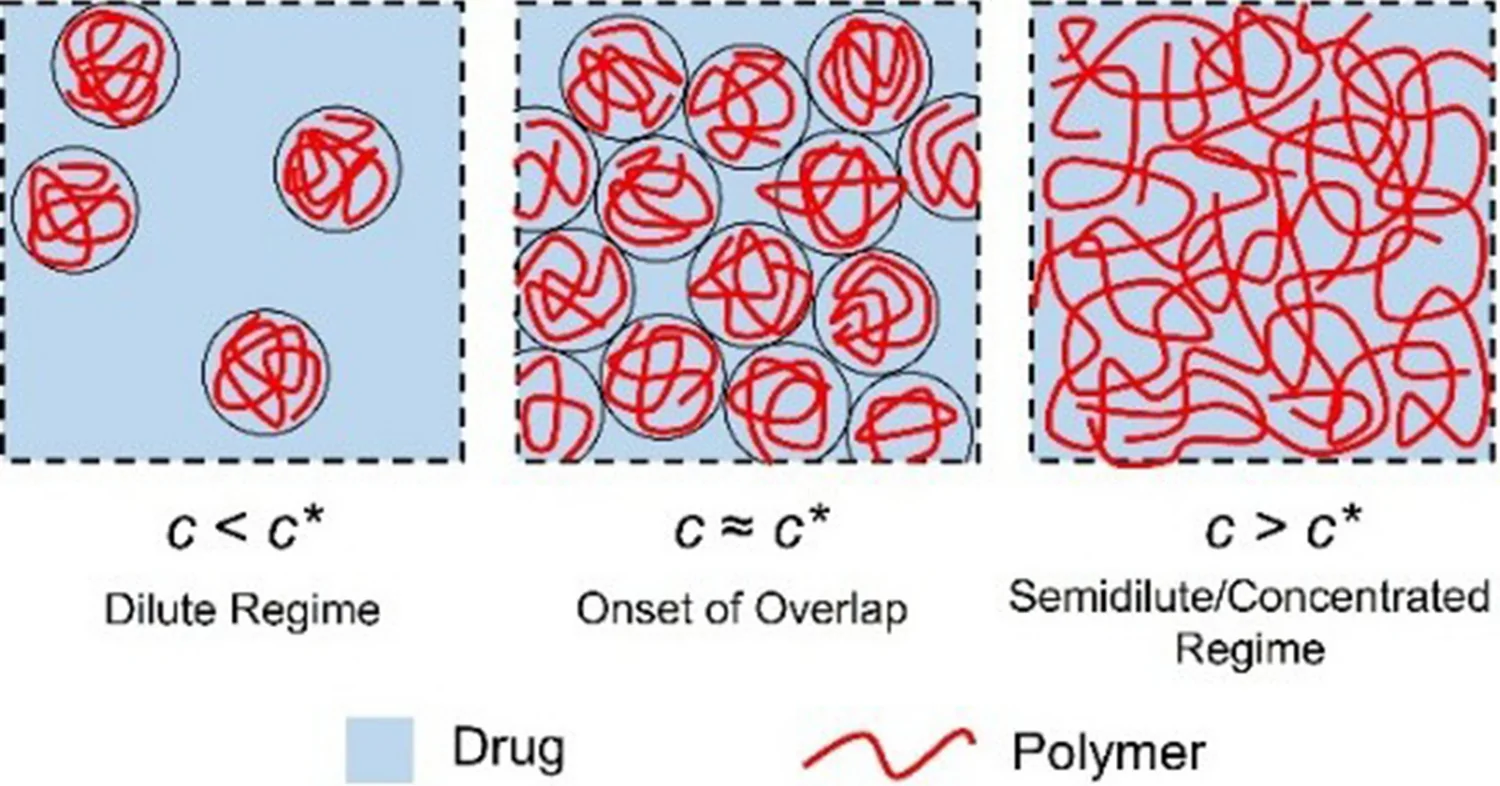

## Introduction

Amorphous solid dispersions (ASDs) are widely used as a formulation strategy to improve the aqueous solubility, dissolution rate, and hence oral bioavailability of poorly soluble drugs [1–5]. A typical binary ASD consists of an amorphous drug dispersed in a polymer at the molecular level. The polymer excipient plays an important role in determining an ASD’s performance, including drug release kinetics, supersaturation maintenance, and physical stability against crystallization during storage [6–8].

One of the primary challenges in ASD development is the intrinsic thermodynamic instability of amorphous materials, which tend to crystallize over time, thereby negating their solubility advantage [7, 8]. Crystallization involves both nucleation and crystal growth, processes that are governed by molecular mobility and local order [9, 10]. Polymers are widely used to inhibit crystallization, and their effects have been attributed to reduction of molecular mobility [11], disruption of molecular packing [12], and specific drug-polymer interactions [13]. However, these factors alone do not fully account for the strong dependence of crystallization behavior on polymer concentration [14] and molecular weight [15], particularly in high drug-loaded systems.

Recent advances suggest that concepts from polymer solution theory provide a useful framework for understanding this dependence. In particular, the overlap concentration c*, the threshold at which adjacent polymer coils begin to interpenetrate, defines a transition in the spatial organization of polymer-in-drug systems [16, 17]. Below c*, the system contains a continuous polymer-free amorphous drug domain that behaves similarly to neat amorphous drug, allowing crystallization to proceed readily. Above c*, this domain is eliminated, and a homogeneous polymer-rich domain is formed, leading to significant suppression of crystallization [18–20].

This review is by no means comprehensive; rather, we emphasize the effect of polymer concentration and molecular weight on crystallization behavior in high drug-loaded systems, with particular focus on the role of the overlap concentration, c*, in governing crystallization in ASDs. Readers interested in broader aspects of amorphous pharmaceutical solids [7, 21, 22], including preparation [23], characterization [24], and general polymer based stabilization strategies in both the solid and solution states, are referred to other comprehensive reviews and references therein [4, 5, 8, 25–28].

## Polymer Overlap Concentration and its Correlation with Stability against Crystallization in ASDs

**Fig. 1:**
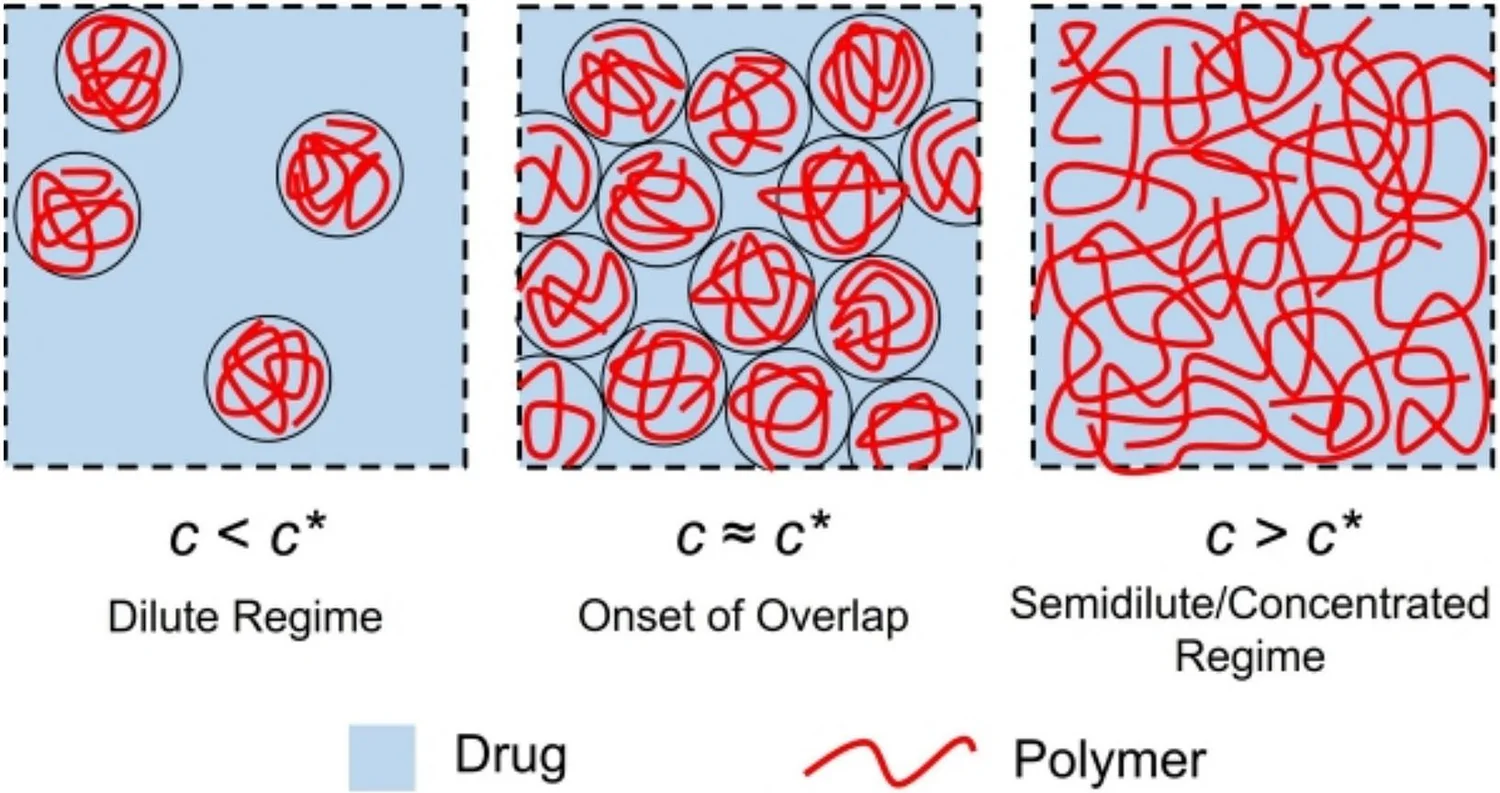
Scheme of the crossover between dilute (left) and semidilute or concentrated (right) polymer-in-drug solutions. The onset of overlap between adjacent polymer coils is c*.

According to polymer solution theory, in a good solvent, which has strong attractive interactions with dissolved polymers over a broad range of temperature, polymer solutions can be roughly categorized into three regimes, namely, dilute, semidilute, and concentrated [16, 17]. In the dilute regime, polymer concentration is sufficiently low that polymer coils are separated (Fig. 1 left). In many cases, the individual coils substantially swell due to their interaction with solvent (in the present case, drug) molecules. The coils do not overlap, however, since the increasing concentration of polymer in the overlapping regions of adjacent coils increases free energy. As polymer concentration increases, polymer coils come closer to each other. At the critical overlap concentration, c*, the coils become densely packed (Fig. 1 middle). Above c*, in the semidilute regime, the coils have no choice but to interpenetrate (Fig. 1 right). Thus, c* serves as the threshold between the dilute and semidilute regimes [16]. In the concentrated regime, polymer chains overlap significantly and revert to Gaussian conformations due to screening of excluded volume interactions [16].

The overlap concentration c* is correlated with the crystallization tendency of ASDs (polymer-in-drug solutions). A dilute ASD consists of two types of domains at the molecular level: (1) discontinuous polymer-rich domains within each isolated coil and (2) a continuous polymer-free amorphous drug domain. The latter effectively behaves as neat amorphous drug, permitting rapid crystallization (see Sect. "Overall Crystallization Behavior" and "The First Nucleation Event"). In contrast, at polymer concentrations above the threshold (*c* > c*), overlap and/or interpenetration of polymer chains eliminates the polymer-free domain. As a result, polymer-mediated crystallization inhibition becomes spatially pervasive, leading to a marked suppression of crystallization tendency throughout the system.

## Overlap Concentration Determination by Rheometry

### Determination of c* from Viscosity-Composition Diagram

**Fig. 2:**
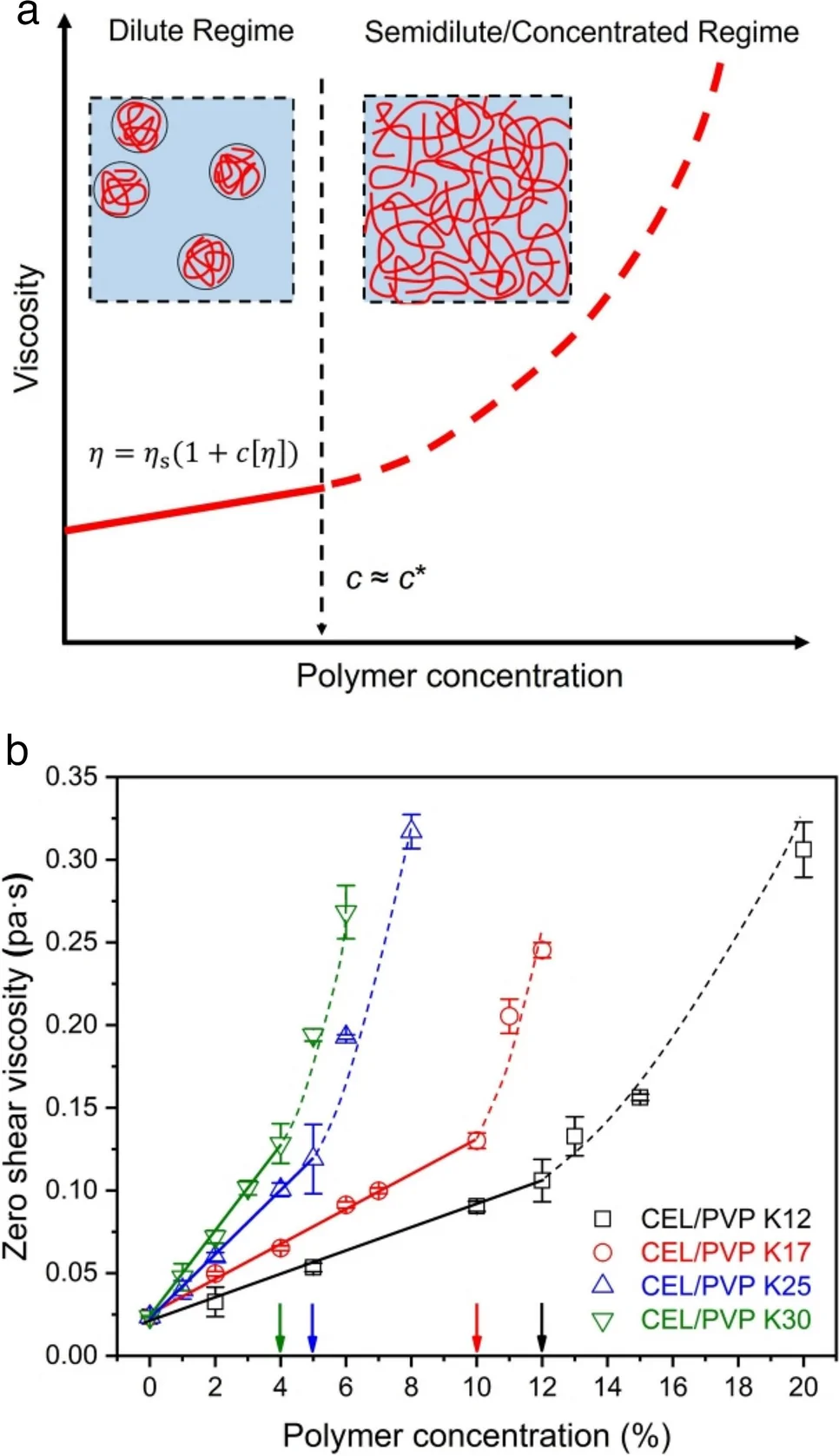
(**a**) Illustration scheme of viscosity of drug-polymer melt change with polymer concentration; (**b**) Viscosity-composition diagram of CEL/PVP measured at 165 ℃. Panel (**b**) reproduced from Ref. [18] with permission. Arrows represent c* estimates.

Polymer solution theory describes the change of overall (zero shear rate) viscosity of polymer solutions as a function of polymer concentration, as illustrated by Fig. 2a. For a dilute solution, since polymer coils are isolated from each other, intermolecular interactions between adjacent coils are negligible and the viscosity *η* is approximately linear with respect to polymer concentration* c*:

1 $$\eta ={\eta}_{\mathrm{s}}\left(1+c{\left[\eta \right]}_{\mathrm{w}}\right)$$

where ${\eta}_{\mathrm{s}}$ is the viscosity of the pure solvent (neat drug melt) and ${\left[\eta \right]}_{\mathrm{w}}$ is the intrinsic viscosity of the polymer/drug combination, expressed in units of weight %^−1^ [18, 29]. It differs slightly from the conventional intrinsic viscosity, $\left[\eta \right]$, in that the latter is calculated based on w/v polymer concentrations and expressed as mL/g.

In the semidilute regime, the onset of overlap among polymer coils introduces coil-coil interactions that contribute additional resistance to flow, resulting in an increased slope of the viscosity-composition curve. Upon entering the concentrated regime, viscosity rises more sharply, partially due to entanglement of long chains, such that neighboring chains impose topological constraints, effectively confining each chain to a tube-like region, thereby substantially increasing the viscosity. However, there is no general nonlinear equation to capture the behavior of the *η* – *c* curve in the semidilute and/or concentrated regimes. The crossover between linear (dilute) and nonlinear regimes (semidilute/concentrated) of a viscosity-composition diagram is identified as c* [17, 18]. However, note that this transition is not sharp as in a critical phenomenon, and c* actually corresponds to a narrow concentration range [16].

Figure 2b shows viscosity-polymer concentration curves for four grades of polyvinylpyrrolidone (PVP), with weight average molecular weight* M*_w_ ranging from approximately 4–55 kg/mol (see Table 1), in the nonsteroidal anti-inflammatory drug (NSAID) and selective COX-2 inhibitor celecoxib (CEL). Measurements were conducted at 165 °C, slightly above the melting point of CEL (*T*_m_ = 160.8 °C for Form III), to guarantee complete melting and obtain reliable viscosity measurements. The values of c* for PVP K12 (*M*_w_ ≈ 4 kg/mol), PVP K17 (*M*_w_ ≈ 7 kg/mol), PVP K25 (*M*_w_ ≈ 50 kg/mol), PVP K30 (*M*_w_ ≈ 55 kg/mol), are approximately 12%, 10%, 5%, 4%, respectively [18, 30]. With molecular weight increase, c* decreases, following the expected scaling relation c* ~ *M*_w_^−0.8^ [16]. For sufficiently high molecular weights, c* is quite small. For example, in linear polymers such as a high grade PVP K90 (*M*_w_ ≈ 1–2 × 10^6^ g/mol), c* is approximately 0.5% w/w, which is challenging to determine precisely in experiments.

**Table 1:** Values of *M*_n_* M*_w_* Ð* of PVPs and comparison of c* values of PVP in CEL determined by methods of viscosity-composition diagram and [*η*]_w_^−1^ with prefactor between 1–2.5 [18]

	*M*_n_ (g/mol)	*M*_w_ (g/mol)	*Ð* (*M*_w_/*M*_n_)	c* by Viscosity – composition diagram (%)	c* by [*η*]_w_^−1^ (%)
PVP K12	2,300	3,790	1.65	12	3.2–8.1
PVP K17	4,050	7,340	1.81	10	2.7–6.7
PVP K25	25,710	49,480	1.92	5	1.2–3.0
PVP K30	30,360	55,090	1.81	4	0.7–1.8

It is worth mentioning that, as exemplified by the PVP grades used above, many pharmaceutical dispersion polymers exhibit relatively broad molecular weight distributions. However, c* appears to be relatively insensitive to polymer polydispersity (*Ð* = *M*_w_/*M*_n_). This observation can be rationalized by recognizing that c* reflects the *average concentration* at which individual polymer coils, each occupying a spherical region with radius of gyration *R*_g_ collectively pervade the entire volume. Consistent with this interpretation, a narrowly dispersed PVP (*M*_w_ = 8,850 g/mol, *Ð* = 1.12) synthesized via reversible addition-fragmentation chain transfer (RAFT) polymerization, exhibits c* ≈ 10% in felodipine. This value is comparable to that of a commercial, more polydisperse PVP of similar molecular weight (*M*_w_ = 8,900 g/mol, *Ð* = 1.99), for which c* ≈ 11% [31].

### Determination of c* from the Inverse of [*η*]

A commonly used scaling relation for estimating c* is ${c}^{*}\cong 1/{\left[\eta \right]}_{\mathrm{w}}$ [31, 32]. This relation is based on the assumption that, at *c* ≈ c*, the contributions of individual polymers and the pure solvent (i.e., the molten drug) to the overall viscosity are comparable. However, this estimation is approximate, and alternative forms incorporating different prefactors have also been proposed, such as ${c}^{*}\cong 2/{\left[\eta \right]}_{\mathrm{w}}$ or ${c}^{*}\cong 2.5/{\left[\eta \right]}_{\mathrm{w}}$ [29]. Theoretically, no approach is optimal as c* is not a particular concentration at which something dramatic happens (i.e., discontinuities in n^th^ order derivatives of the free energy associated with an n^th^ order phase transition [29]), and hence there is no consensus on the theory of c*. Table 1 compares c* values of CEL/PVP combinations determined by viscosity-composition diagrams and by [*η*]_w_^−1^ with prefactor between 1–2.5. The c* values obtained from viscosity-composition diagrams are approximately 2–3 times higher than those estimated from the scaling relation c* ~ 1/[*η*]_w_, reflecting that addition of PVP contributes disproportionately to the viscosity, by roughly a factor of four, relative to neat CEL. While there is no definitive theoretical basis to distinguish the accuracy of these estimates, the c* values derived from viscosity-composition diagrams for CEL/PVP systems show a stronger correlation with crystallization tendency (see Sect. "Overall Crystallization Behavior"). Because of the theoretical uncertainty regarding the scalar factor relating c* to 1/[*η*]_w_, and the relatively tighter estimates of c* that can be obtained from visual examination of the viscosity *versus* polymer concentration plots, we have chosen the latter method for the most recent and future studies.

**Fig. 3:**
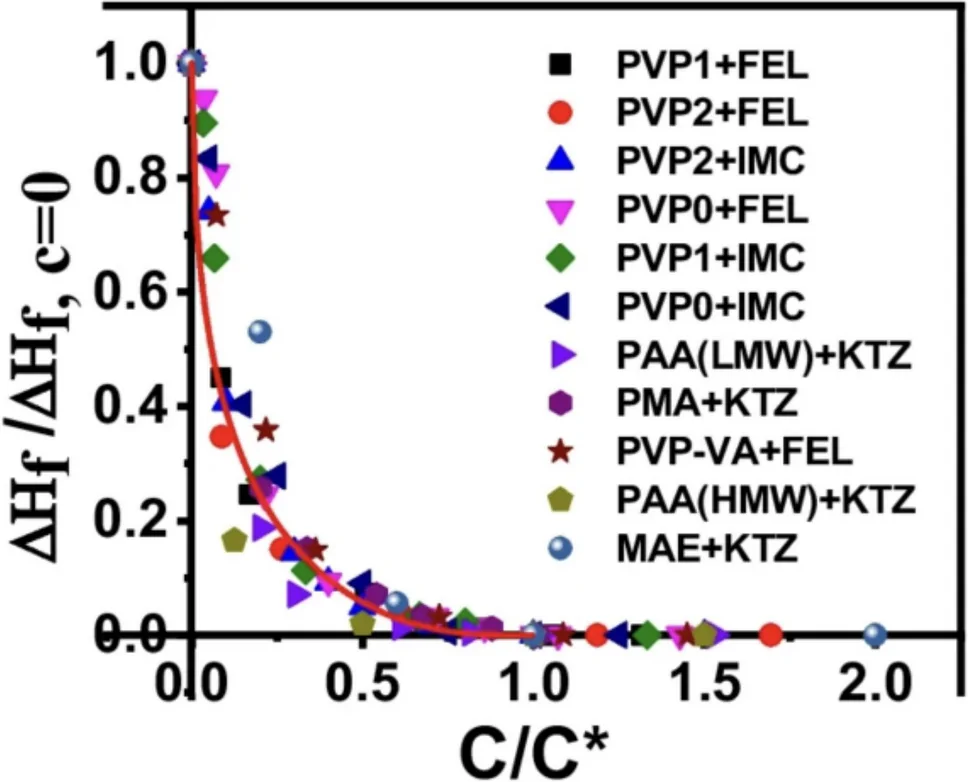
Normalized heats of fusion against *c*/c* of various drug-polymer combinations. Reproduced from Ref. [32].

However, before making the latter decision, we conducted experiments with a variety of drugs and polymers using the ${c}^{*}=1/{\left[\eta \right]}_{\mathrm{w}}$ supposition [31, 32]. Figure 3 shows the results of these studies, in which we plot the ratios of the normalized enthalpies of fusion of fresh ASDs to their corresponding values for neat drug, as determined by differential scanning calorimetry with a heating rate of 10 °C/min, *versus* polymer concentration, *c*, normalized by the intrinsic viscosity-based estimate of c*. Interestingly, the results “collapse” to a master curve, with the enthalpy ratio decreasing with *c*/c*, until it reaches zero near *c*/c* = 1. The uncertainty in c* indicates that the plotted *c*/c* values could be subject to lateral shifting, mostly to the left. Nevertheless, it is clear that when *c* exceeds c*, there is no heat of fusion upon addition of polymer, signaling the absence of crystal formation in the ASDs upon heating.

For *c* < c*, the data are reasonably well fitted (Fig. 3) by the function

2 $$\frac{\Delta {H}_{\mathrm{f}}}{\Delta {H}_{\mathrm{f},c=0} }={e}^{{-3.66\left(\frac{c/c*}{1-c/c*}\right)}^{0.66}}$$

which might be considered as a variation of free volume theory [32]. Here the “free volume” is identified with the polymer-free region, and the drug-swollen polymer chains are regarded as hard spheres that exclude nucleation and growth of amorphous drug. Before overstating the analogy, however, it should be noted that the exponent 0.66 does not appear in classical free volume theory [33]. Of course, improving the precision of the above relation is of little practical interest, since the formulator is interested only in cases where there is no crystallization, i.e., *c* > c*.

### Impact of Temperature and Processing Methods on the Determination of c*

The overlap concentration c*, determined from the high-temperature rheological approach as discussed above, is intrinsically defined in the molten state (i.e., slightly above the drug melting point *T*ₘ), where both the drug and polymer chains are fully equilibrated and processing history is effectively erased. This raises two critical considerations: (1) whether c* is temperature-dependent when extrapolated to a low storage temperature (i.e., in a deeply cooled state below *T*_g_), and (2) whether c* remains valid across different manufacturing routes.

The temperature dependence of c* can be assessed by comparing the polymer concentration threshold at which pure amorphous drug and polymer rich domains merge (c*), as determined in the molten state, with that measured in glassy ASDs. Solid-state NMR (ssNMR) spectroscopy has been widely used to evaluate miscibility and probe spatial heterogeneity in multicomponent systems [34–37], with ^1^H T_1ρ_ measurements sensitive to ~ 2–5 nm, and ^1^H T_1_ measurements to ~ 20–50 nm length scales, comparable to the spherical region radius *R*_g_ of typical dispersion polymers [36, 38, 39]. For nifedipine(NIF)/PVP K12 ASDs, where *R*_g_ of PVP K12 (≈ 4 nm) is comparable to the length scale probed by the ^1^H T_1ρ_ method, the spatial heterogeneity transition observed by ssNMR in the glassy state at −20 ℃ occurs at a polymer concentration consistent with the c* value of ~ 18% determined from high-temperature rheology at 175 ℃. Below c*, distinct ^1^H T_1ρ_ relaxation times indicate the coexistence of pure amorphous NIF and PVP-rich domains, whereas above c*, the convergence of ∆^1^H T_1ρ_ reflects homogeneous mixing at the molecular level [39]. This agreement suggests that the structural condition defining c* is largely preserved upon rapid vitrification.

The influence of processing methods on the determination of c* has been examined by comparing ASDs prepared via melt quenching *versus* spray drying [30]. While the rheological approach characterizes ASDs in the molten state and thus removes processing history, the nondestructive ssNMR method probes the as-prepared ASD materials and retains information on processing-induced heterogeneity. For CEL/PVP K17 ASDs, ssNMR measurements show that the evolution of spatial heterogeneity with polymer concentration is largely consistent between melt-quenched and spray-dried samples, with the transition occurring near c* ≈ 10% [30]. Below c*, both systems exhibit distinct ^1^H T_1ρ_ relaxation times, indicating heterogeneous structures, while above c*, the convergence of relaxation times reflects molecular-level mixing. Minor differences are observed at intermediate compositions, where spray-dried dispersions exhibit slightly greater heterogeneity, likely due to surface compositional heterogeneity (e.g., polymer enrichment or depletion) arising during processing [40, 41]. Nevertheless, the consistency in the miscibility/spatial heterogeneity threshold and corresponding crystallization behavior indicates that c* captures an intrinsic structural feature that is largely independent of processing route.

## Crystallization Kinetics

### Overall Crystallization Behavior

**Fig. 4:**
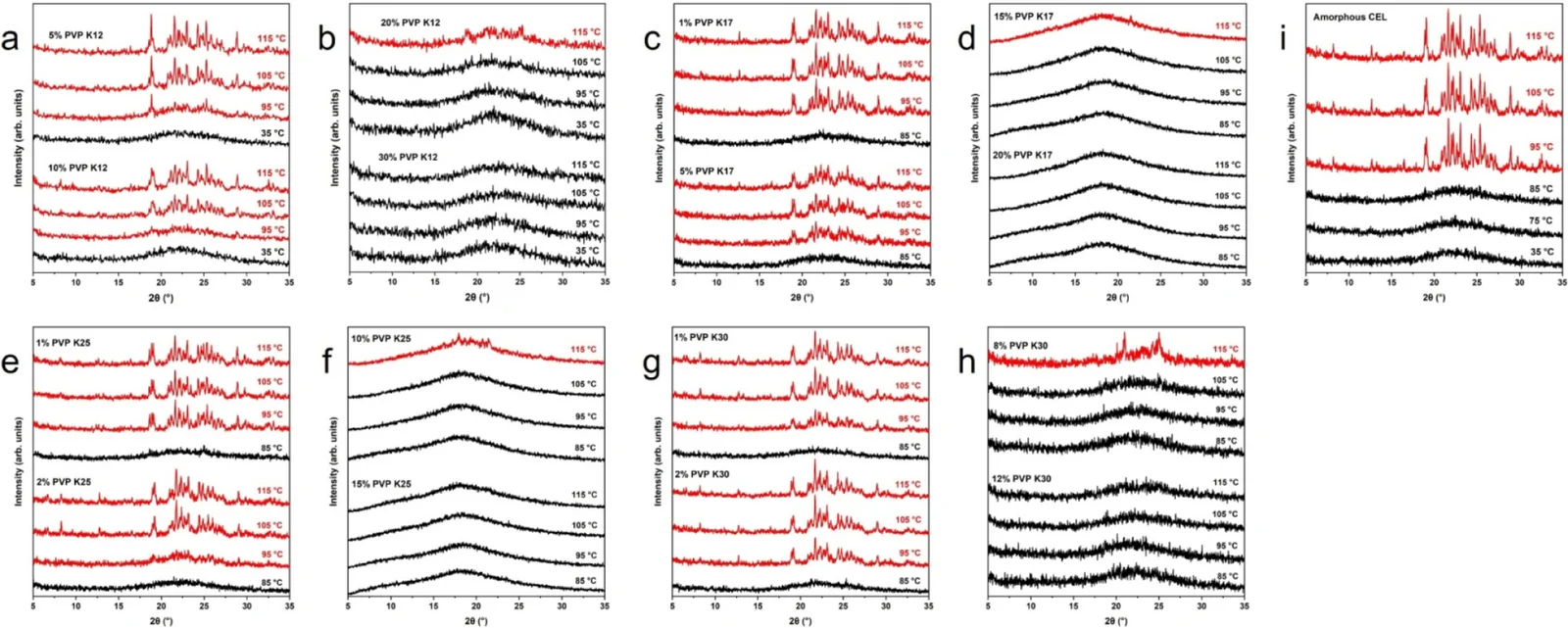
XRD patterns of melt-quenched CEL/PVP (K12, K17, K25, K30) ASDs with various polymer concentrations below and above c* subjected to a controlled temperature program under 0% RH. The temperatures at which the patterns were obtained are indicated. Adapted from Refs. [18, 30] with permission.

Crystallization kinetics of ASDs are governed by crystal nucleation and growth, both of which are strongly modulated by polymer concentration and spatial distribution [42, 43]. When *c* < *c*^⁎^, the presence of the pure amorphous drug domain enables crystallization to proceed comparable to that of neat amorphous drug, resulting in a limited inhibitory effect. In contrast, when *c* > *c*^⁎^, the formation of a homogeneous, polymer-rich matrix suppresses both nucleation and growth by reducing molecular mobility and disrupting the structural organization required for crystallization. Consequently, *c*^⁎^ serves as a critical threshold that delineates regimes of rapid *versus* effectively inhibited crystallization in ASDs. This view is supported by accelerated stability studies of CEL/PVP ASDs using variable temperature X-ray diffractometry. Freshly prepared CEL ASDs with PVP concentrations below and above c* were evaluated from 35 ℃ up to 115 ℃ in 10 ℃ increments. As shown in Fig. 4, for all four PVP grades (K12, K17, K25, K30), tested, dilute ASDs with *c* < *c*^⁎^ exhibit identical crystallization onset temperature of 95 ℃ (panels a, c, e, g), matching that of the neat amorphous CEL (panel i). In contrast, semidilute and concentrated ASDs with *c* > *c*^⁎^ show substantially reduced crystallization tendencies, with onset temperatures elevated to at least 115 ℃ or higher, as shown in panels b, d, f, h [18, 30].

**Fig. 5:**
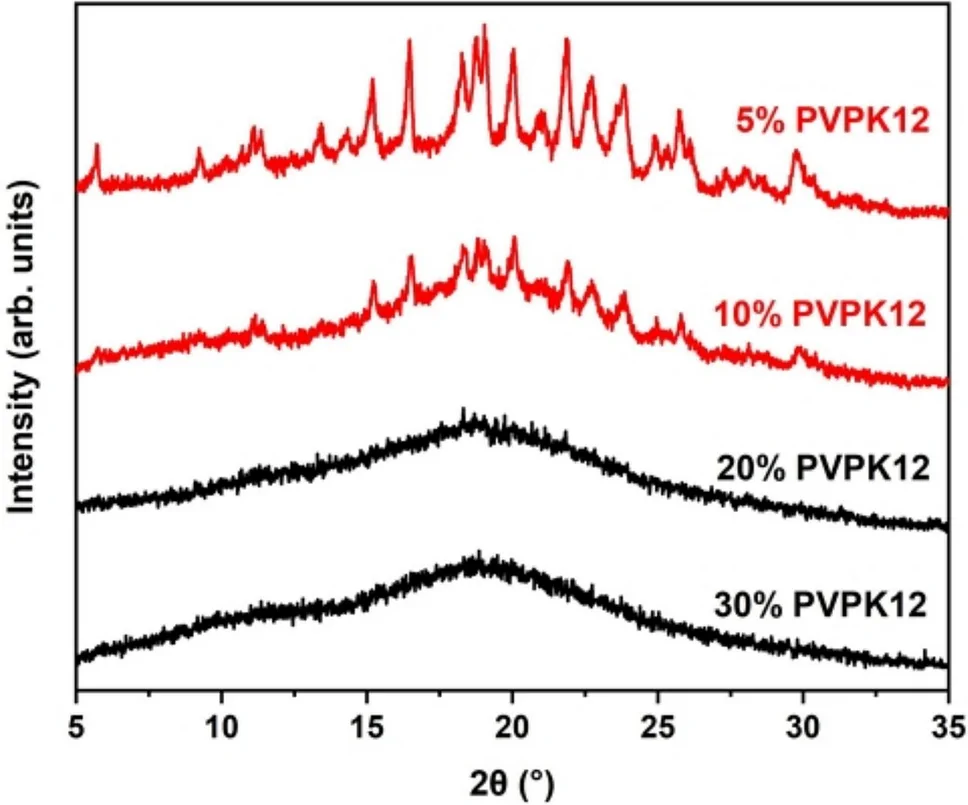
XRD patterns of CEL/PVP K12 ASDs with various polymer concentrations stored under ambient conditions for one year. Reproduced from Ref. [18] with permission.

Figure 5 provides further compelling evidence of the impact of *c*^⁎^ on crystallization behavior in CEL/PVP K12 ASDs (*c*^⁎^ ≈ 12%) under ambient storage over one year. For ASDs with polymer concentrations of 20% and 30% (*c* > *c*^⁎^), no detectable crystallization is observed. In contrast, dilute ASDs containing 5% and 10% PVP (*c* < *c*^⁎^) exhibit pronounced crystallization [18]. These results demonstrate that a *c*^⁎^-guided framework offers reliable predictive capability for long term stability against crystallization, supporting its practical utility in high drug loaded ASD formulation development.

**Fig. 6:**
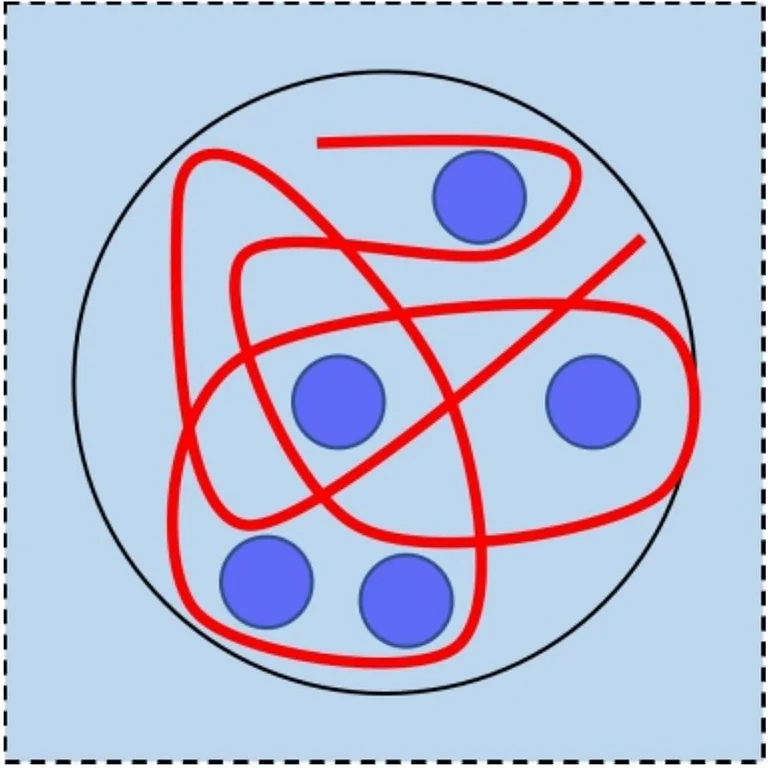
Schematic illustration of an isolated, high molecular weight polymer coil. The low local segment density within the coil permits formation of crystals (bright blue circles).

We close this section by noting that the theory regarding c* as a threshold for suppression of crystallization should not hold for polymers with sufficiently high molecular weight, e.g., *M*_w_ > 1,000 kg/mol, such as PVP K90. In these systems, the local polymer segment density within individual, highly expanded coils remains relatively low, leaving sufficient free space for crystallization to occur even when *c* > *c*^⁎^, as illustrated in Fig. 6.

### The First Nucleation Event

Crystallization occurs by nucleation and crystal growth, each exhibiting distinct kinetics [9]. It has been suggested that crystallization inhibition in ASDs by polymers above *c*^⁎^ is strongly correlated with the delay of the first nucleation event [19, 20]. The first nucleation time *t*_0_ is defined as the time required for the formation of the first group of critical nuclei in a fresh ASD at a specific temperature.

**Fig. 7:**
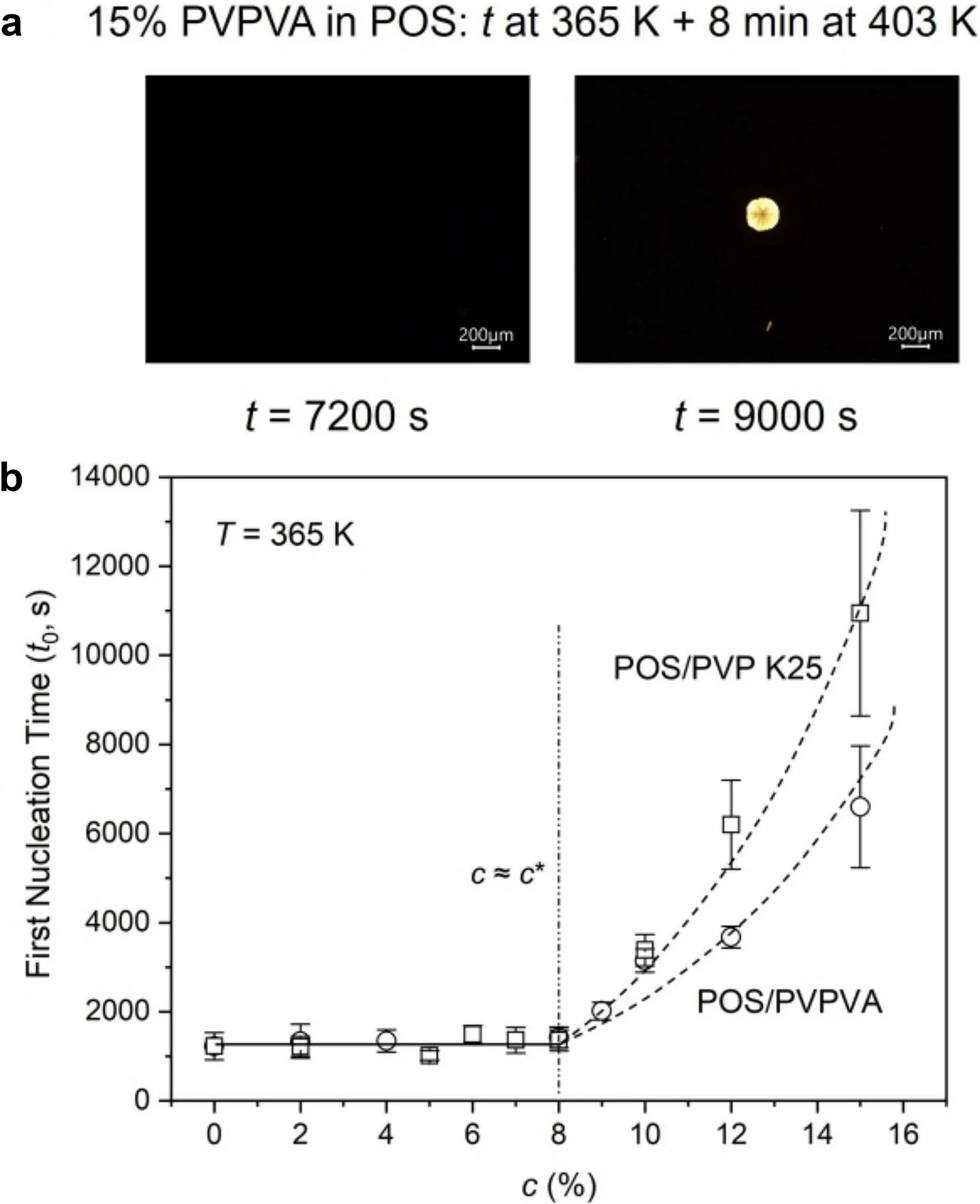
(**a**) The first crystal observed after POS in the presence of 15% PVPVA spent different times at 365 K (7200 or 9000 s) and then 8 min at 403 K to grow. Before heating to 403 K, no crystals were observed. (**b**) First nucleation time of POS/PVPVA and POS/PVP K25 as a function of polymer concentration at 365 K. Dashed curves are drawn to follow trends of increased first nucleation times with increasing polymer concentration. Reproduced from Ref. [20] with permission.

Figure 7a presents representative data for determining *t*_0_ of a fast nucleating, slow growing system, posaconazole (POS) [20]. Amorphous POS/polymer thin films were held at 365 K for a defined period (first stage) to permit nucleation. The temperature was then increased to 403 K (second stage) to promote growth of preexisting nuclei into visible crystals under light microscopy without further nucleation. This procedure was repeated with progressively shorter first stage hold times until no visible crystals were observed in the second stage. The value of *t*_0_ was defined as the average of the last two consecutive hold times bracketing this transition.

**Fig. 8:**
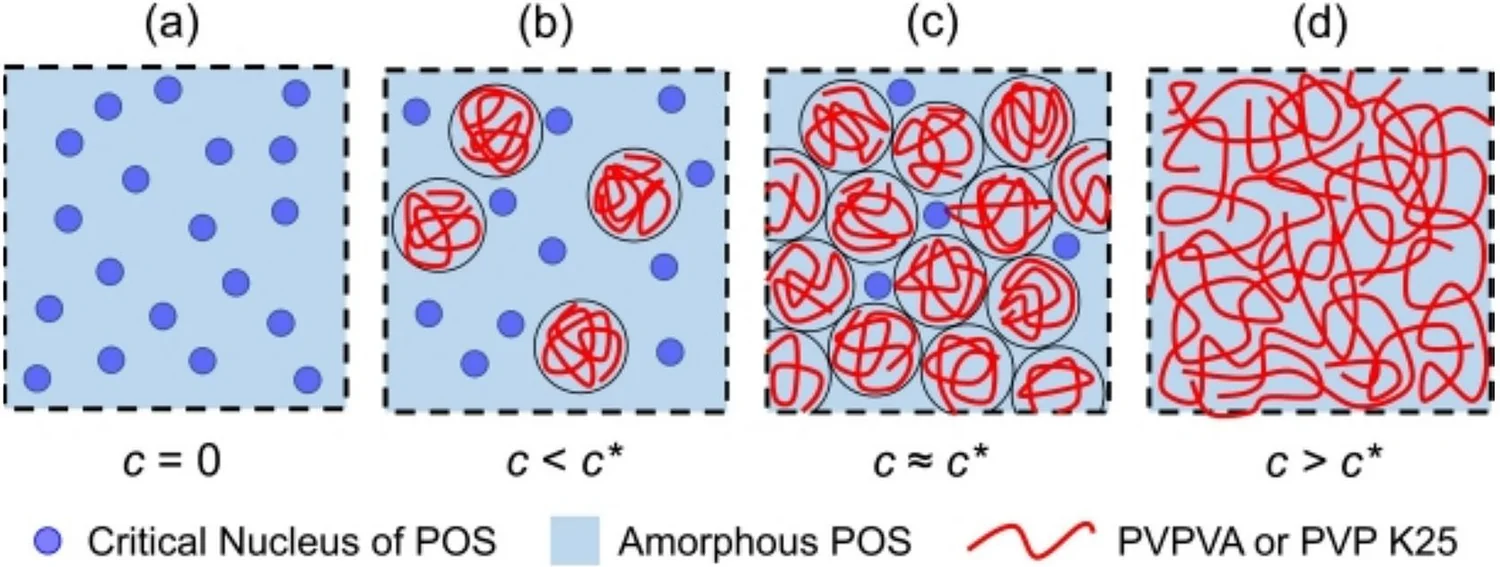
Illustration of the delay of the first nucleation event in semidilute/concentrated (**c**-**d**) polymer solutions compared with that in neat amorphous drug (**a**) and a dilute solution (**b**). Reproduced from Ref. [20] with permission.

Figure 7b compares *t*_0_ values for POS/PVP K25 and POS/PVPVA films with polymer concentrations below and above *c*^⁎^ (8% for both polymers). When *c* ≤ *c*^⁎^, the *t*_0_ values for both POS/PVP K25 and POS/PVPVA were close to identical with that of neat amorphous POS, indicating negligible delay of the first nucleation event. In contrast, when *c* > *c*^⁎^, *t*_0_ increased progressively with polymer concentration, reflecting a growing suppression of nucleation kinetics, as depicted in Fig. 8 [20].

A similar trend where the value of *t*_0_ remains identical to that of the neat amorphous solid when *c* ≤ *c*^⁎^, but increases substantially when *c* > *c*^⁎^ was also observed in a fast nucleating, fast growing system, D-sorbitol, with PVP K25 and K30 [19]. In this case, *t*_0_ was determined using a single stage method, where the first nucleation time was estimated by subtracting the growth period from the total observation time based on tracking the emergence of the largest spherulites.

The above observations, as illustrated in Fig. 8, depend on an intermediate polymer molecular weight, more specifically on a characteristic size ratio between the polymer coil radius *R*_g_ and the critical nucleus size *r*_c_ being on the order of ~10. Given that *r*_c_ is typically ~ 1 nm for many molecular systems, this condition corresponds to polymers with *M*_w_ ≈ 10–100 kg/mol. For low molecular weight polymers, where *R*_g_ is comparable to *r*_c_, the first nucleation time *t*_0_ begins to increase at polymer concentrations slightly below *c*^⁎^. For example, *t*_0_ for D-sorbitol with PVP K12 (*M*_w_ ≈ 4 kg/mol) and PVP K17 (*M*_w_ ≈ 7 kg/mol) increases at 0.625 *c*^⁎^ and 0.667 *c*^⁎^, respectively [19]. However, for high molecular weight polymers such as PVP K90 (*M*_w_ ≈ 1,500 kg/mol), where *r*_c_ ≪ *R*_g_ (Fig. 6, in which bright blue circles represent critical nuclei), effective inhibition of the first nucleation event is achieved only at concentrations well above *c*^⁎^.

### Crystal Nucleation and Growth Rates below and above c*

**Fig. 9:**
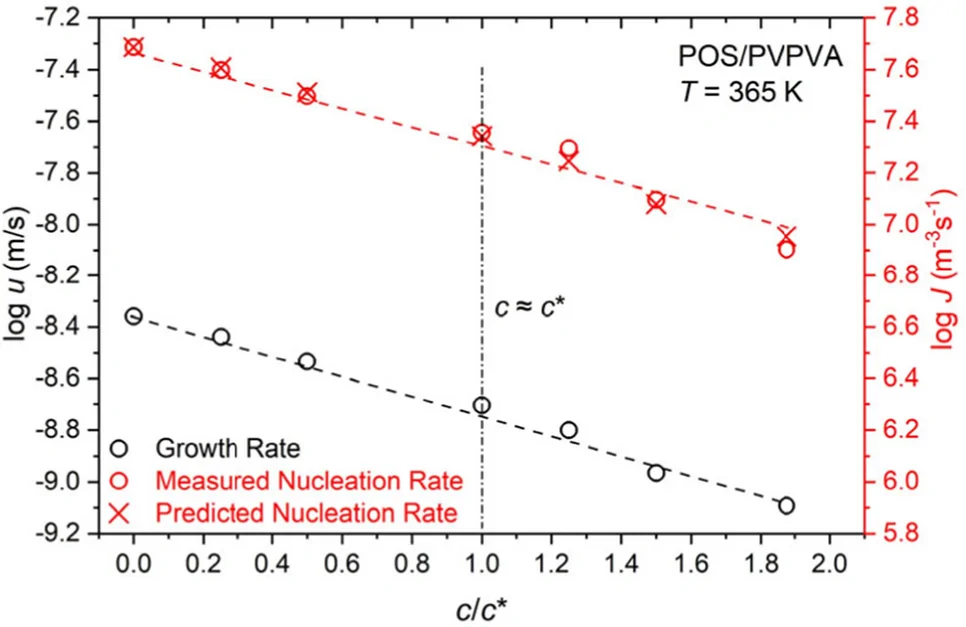
Effect of PVPVA concentration on the steady state rates of crystal nucleation *J* and growth *u* in POS at 365 K. Reproduced from Ref. [20] with permission.

The exclusive role of *c*^⁎^ in delaying the first nucleation event has been further examined by evaluating its influence on the steady state rates of nucleation *J* and crystal growth *u*. Figure 9 shows the dependence *J* and* u* on polymer concentration in POS at 365 K across *c*^⁎^. With increasing PVPVA concentration, both log *J* and log *u* decrease linearly to a similar extent, following log (*J*/*u*) ≈ 16.0 m^−4^, as both processes share the same kinetic factor and exhibit similar molecular motions [9, 10, 42].

Notably this smooth, continuous dependence of *J* and *u* spans the low concentration regime and passes through *c*^⁎^ without any discontinuity. Similar behavior is observed in D-sorbitol/PVP combinations (K12, K17, K25, K30) and D-arabitol/PVP K30, [42] assuming a comparable* c*^⁎^ value. These results indicate that the pronounced suppression of crystallization above *c*^⁎^ is primarily correlated with the delay in the first nucleation event, rather than abrupt changes in the steady state nucleation and crystal growth rates.

## Discussion and Concluding Remarks

We have reviewed recent advances in understanding the role of polymer concentration, particularly the overlap concentration c*, in governing crystallization in amorphous drug-polymer dispersions. When polymer concentration *c* ≤ *c*^⁎^, the presence of the continuous, polymer-free amorphous drug domain results in minimal inhibition, and the overall accelerated crystallization tendency remains comparable to that of neat amorphous drug. In contrast, when *c* > *c*^⁎^, elimination of the polymer-free domain and formation of a homogeneous, polymer rich matrix lead to substantial suppression of crystallization. Importantly, this inhibitory effect is strongly correlated with delay in the first nucleation event, rather than abrupt changes in steady state nucleation and crystal growth rates. These insights provide a mechanistic framework for rational polymer selection and optimization of performance in high drug loaded ASD formulations.

Almost all prior theoretical approaches to estimating the polymer concentration needed to stabilize amorphous drugs have been based Flory–Huggins (FH) theory, which requires determination of the polymer/drug interaction parameter, *χ*, or perturbed chain statistical associated fluid theory (PC-SAFT), which utilizes predetermined molecular group parameters to estimate polymer/drug interactions [44]. Both approaches take the view of drug being dissolved in polymer, with the latter usually being the majority component. These theories predict the thermodynamic equilibrium solubility of drug in polymer, and predict that higher drug concentrations would lead, at equilibrium, to a phase containing drug crystals. In contrast, our approach is based on the fact that drug crystals require nucleation to form, and the hypothesis that nucleation can be suppressed when the critical nucleus diameter is greater than the characteristic distance between polymer coils. For intermediate molecular weight polymers, this criterion is met when *c* > c* (Fig. 8) while for very high molecular weight polymers, the characteristic distance of interest is the monomer–monomer correlation length (Fig. 6). Such large polymers are difficult to process, however, so they are not of much interest for ASDs. It should also be noted, with respect to FH theory, that polymer/drug *χ* parameters have been estimated by melting point depression of drug crystals in the presence of polymer, which is measured at temperatures that are far above the temperature of storage. The formulas used to compensate for difference in temperature are likely to be invalid for such large differences, as they do not take into account the effects of heat capacity [45]. Large and uncertain negative reported values of *χ*, which are likely to be due to strong interactions such as hydrogen bonding between drug molecules and polymer segments, and which call into question the random mixing entropy assumption, also contribute to doubt regarding the appropriateness of FH [46].

The approach reviewed here relies only on empirical determination of c*, which is taken as the only parameter. It also views polymer as “solute” and drug as “solvent”. No molecular theory is needed, although such theories could be adapted to predicting c*. We speculate that the stiffness of the polymer chains, as reflected in the persistence length, will play a role, especially for cellulose-based polymers. For such stiff polymers, the situation illustrated in Fig. 6 may occur at relatively low polymer concentrations.

One possible direction for future work is optimization of methodologies for determining c*. Current approaches, either by locating the crossover between linear and nonlinear regimes in viscosity-composition diagram or extrapolating from the scaling relation c* ~ 1/[*η*] require relatively large sample quantities (~ 800 mg per measurement) to obtain reliable zero shear viscosity data across multiple compositions. Developing alternative methods that reduce material requirements (e.g., to ~ 5 mg) would be highly advantageous. One potential strategy is to infer viscosity from surface grating decay kinetics using Mullins’s model [47, 48], which could enable measurements with minimal sample consumption of ~ 2 mg per measurement. This is particularly relevant for early-stage development, where material availability is often limited.

A second direction of potential future work is to assess the generality of the c*-guided framework across a broader range of drug-polymer combinations. While some non-vinylpyrrolidone polymers, such as polyacrylic acid and polymethacrylic acid have been examined [32], the majority of existing studies focus on vinylpyrrolidone based polymers. Systematic evaluation of polymers with diverse chemical structures and physicochemical properties would help establish the broader applicability and limitations of this framework. In particular, studies should ascertain any effects of polymer *T*_g_, which are completely ignored in our c*-guided approach. The polymers used to date have *T*_g_s that are above those of the drugs, and most authors assume that this contributes to the polymer’s effect on drug molecular mobility and hence its crystallization kinetics. It would be interesting to see whether the c* approach applies to ASDs with polymer additives exhibiting a variety of *T*_g_s [49]. In a similar vein, all experiments reported so far have been carried out with essentially dry samples. The influence of humidity should be studied, since the presence of water can, even at low levels, increase the mobility of drug and polymer. As low water content in an ASD would have little effect on c*, the present approach predicts negligible water effect.

We close this contribution with a caveat—we have only been concerned with solid state stability against crystallization during storage of ASD formulations. However, a wealth of literature has demonstrated that in many cases, higher drug load ASDs show slow release of both drug and polymer, whereby the drug release rate is comparable to that of the neat amorphous drug [50]. Indeed, we recently observed that dissolution of POS formulated with HPMCAS incorporated at c* (3%) shows poor drug release, and that the polymer concentration in the ASD had to be increased substantially to promote rapid drug release [51]. It should be noted however that POS is a fast crystallizer, which may account for its rapid precipitation. A more extensive study of ASDs containing fast *versus* slow crystallizers could resolve this issue. Further, as a BCS Class II drug, POS’s rapid *in vivo* absorption following release may compete with crystallization. This aspect is not well modeled by the closed systems used to assess drug release. The situation might be better recapitulated by a recently developed Artificial Gut Simulator [52], which allows material sparing physical simulation of simultaneous dissolution and absorption, with tunable absorption rate constant.
